# Proenkephalin a 119–159 (penKid) – a novel biomarker for acute kidney injury in sepsis: an observational study

**DOI:** 10.1186/s12873-019-0283-9

**Published:** 2019-11-28

**Authors:** Mari Rosenqvist, Kevin Bronton, Oliver Hartmann, Andreas Bergmann, Joachim Struck, Olle Melander

**Affiliations:** 1 Department of Clinical Sciences, Lund University, Jan Waldenströms gata 35, 214 28 Malmö, Sweden; 2 Infectious Disease Unit, Skåne University Hospital, Malmö, Sweden; 3 Sphingotec GmbH, Neuendorfstraße 15A, Hennigsdorf, 16761 Germany; 4 Department of Internal Medicine, Skåne University Hospital, Malmö, Sweden

**Keywords:** Pro-enkephalin, penKid, Sepsis, Acute kidney injury, Emergency department, AKI

## Abstract

**Background:**

Sepsis is a leading cause of death worldwide and a major challenge for physicians to predict and manage. Proenkephalin A 119–159 (penKid) is a reliable surrogate marker for the more unstable endogenous opioid peptide enkephalin, which has previously been shown to predict both acute and chronic kidney disease. The aim of this prospective observational study was to assess penKid as a predictor of acute kidney injury (AKI), multi-organ failure and mortality in sepsis among unselected sepsis patients presenting to the emergency department (ED).

**Method:**

We enrolled 644 patients consecutively during office-hours (6 AM-6 PM) between December 1, 2013 and February 1, 2015. Fifty-six patients were excluded due to incomplete data. We measured penKid in 588 adult patients (patients under 18 years of age were excluded) with sepsis (≥2SIRS criteria + suspected infection) upon admission to the ED at Skåne University Hospital, Malmö, Sweden. Logistic regression analysis was used to relate levels of penKid at presentation to AKI, multi-organ failure, 28-day mortality and progression of renal SOFA subscore. Odds ratios are presented as the number of standard deviations from the mean of log-transformed penKid.

**Results:**

In age and sex adjusted models, penKid predicted AKI within 48 h and 7 days, but these associations were attenuated after additional adjustment for estimated creatinine-based glomerular filtration rate (eGFR). In models adjusted for age, sex and eGFR, penKid significantly predicted progression from rSOFA = 0 and ≤ 1 to higher rSOFA scores as well as multi-organ failure and mortality. In contrast, eGFR did not predict 28-day mortality.

**Conclusion:**

PenKid is an effective predictor of renal injury, severe multi-organ failure and mortality in unselected sepsis patients presenting to the emergency department.

## Background

Sepsis is a condition defined by life-threatening organ dysfunction due to a dysregulated host response to infection [1]. The renal system is particularly susceptible as one in two patients with septic shock develop acute kidney injury (AKI) and are at increased risk of both severe morbidity and higher mortality [2–5]. Serum creatinine (SCr) is the established biomarker of kidney function in current practice and defines AKI (KDIGO & RIFLE criteria) [6]. Septic AKI (or sepsis associated AKI or AKI in sepsis) is a syndrome characterized by concomitant bacteremia, azotemia and oliguria [4].

### Importance

The suitability of SCr as a cornerstone marker for septic AKI is not unquestioned. The influence of non-renal factors, such as creatinine metabolism and fluid balance, on SCr concentration has prompted calls for new and more reliable biomarkers for AKI in sepsis [7, 8].

Proenkephalin A 119–159 (penKid) is a reliable surrogate marker for the more unstable endogenous opioid peptide enkephalin [9]. Enkephalins are known to be involved in various physiological processes by binding opioid receptors [9]. Among these are gamma-opioid receptors, which are expressed in many tissues in the body, but in particularly high density in renal tissue [9].

PenKid has been shown to independently predict a variety of significant conditions related to increased morbidity and mortality, such as incidence of chronic kidney disease in the general population [10], worsening renal function and need for renal replacement therapy (RRT) [11], worsening renal function in acute and congestive heart failure [12] and AKI as a complication to cardiac surgery [13, 14]. In recent studies on septic patients in the intensive care unit (ICU) setting [15–17], it has been shown that penKid is a highly specific biomarker for renal function and associated with AKI. Also, in contrast to other novel biomarkers for AKI prediction, it has been shown that penKid remains highly specific for renal function despite massive inflammatory drive, as concentrations remain low in the absence of renal dysfunction in the septic patient [15].

### Aim

In this study, we assess penKid as a biomarker for renal function and its suitability as a predictor of sepsis related AKI and mortality among unselected patients with sepsis presenting to the emergency department (ED).

## Methods

### Study design and setting

This observational study was conducted at the ED of Skåne University Hospital, Malmö, a tertiary academic center that provides most clinical services, except thoracic and neuro surgery. The hospital has 600 beds and serves a population of about 350,000 people, attending to approximately 85,000 visits per year.

The inclusion criteria were suspected infection, as judged by the attending nurse, and meeting of two or more SIRS-criteria (Systemic Inflammatory Response Syndrome, explained below).

### Patient population and data collection

Six hundred and forty-seven patients meeting the inclusion criteria were enrolled between December 1, 2013 and February 1, 2015. Being under 18 years of age was the only exclusion criterion. SIRS was defined as: (a) either body temperature of more than 38 °C, less than 36 °C or self-reported fever/chills within the past 24 h, (b) respiration rate of more than 20 breaths/min and (c) a heart rate of more than 90 beats/min. White blood cell count (WBC count), was not used as an inclusion criterion due to unavailability of lab results during triage. After reviewing the dataset 59 patients were excluded due to incomplete data leaving 588 patients for analysis.

Recruitment was done by an attending research nurse who enrolled patients during office hours (6 A. M to 6 P.M.). Patient medical records were systematically reviewed for demographics, comorbidities and current medications. Information on non-specific supportive therapy, such as supplemental oxygen, intravenous fluids, vasopressor agents, mechanical ventilation, RRT, length of stay, level of care, in-hospital mortality and 28-day all-cause mortality, were recorded. Routine laboratory tests [hemoglobin, WBC count, platelet count, C-reactive protein, SCr, serum bilirubin, serum lactate, activated partial thromboplastin time (aPTT) and International Normalized Ratio (INR)] were analyzed by the local certified laboratory at the Department of Clinical Chemistry of Skåne University Hospital. Both microbiological tests and radiological examinations were noted. EDTA plasma samples were drawn within an hour of presentation to the ED and then frozen within two hours of collection and stored at − 80 °C until analysis. PenKid was measured in duplicates using chemiluminescence immunoassay (Sphingotec GmbH, Hennigsdorf, Germany) at a lab located in Germany by professionals blinded to clinical data in June 2018. Estimated glomerular filtration rate (eGFR) was determined by the formula derived from the Modification of Diet in Renal Disease (MDRD) Study [18].

### Definition of outcomes

After the attending nurse had completed web-based data registration of patient profile and medical records, including lab demographics, comorbidities, ‘limitation of care’-order (regarding cardiopulmonary resuscitation, intensive and ventilator care or any combination of the three), level of care, length of hospital stay, the study physician assessed the presence of organ dysfunction and infection status for each patient. For patients in which assessment was complicated, another two independent specialists of infectious diseases were asked to review the subject data and set a definitive classification. The primary outcomes of this study were AKI development, defined as per AKIN [6] stage 3 (SCr increase of > 44 μmol/L (> 0.5 mg/dL) between any two measurements or need for acute RRT) or an increase in creatinine corresponding to 1.5-fold of baseline with an initial value of > 160 μmol/L (> 2.0 mg/dL) within either 48 h or 7 days. Secondary outcomes were identified as severe multi-organ failure (MOF), defined as four or more failing organ systems (specified below), and 28-day all-cause mortality.

The criteria for organ dysfunction were adopted from the consensus criteria [19] and, at the time, current SSC guidelines i.e. severe sepsis was defined as suspected or confirmed infectious disease in combination with at least two SIRS criteria and presence or development of hypotension, hypoperfusion or organ failure within 48 h after presentation at the ED. [20, 21] Organ failure was stated if any of the following criteria were met: (a) acute neurological dysfunction - patient developed confusion, drowsiness or loss of consciousness; (b) cardiovascular dysfunction – recorded systolic blood pressure of < 90 mmHg, mean arterial pressure < 70 mmHg, decrease in systolic blood pressure greater than 40 mmHg, or the need for vasopressors to maintain blood pressure; (c) respiratory dysfunction – recorded SaO_2_ < 90% or need for mechanical ventilation; (d) renal dysfunction (see AKI above); (e) hematologic dysfunction - platelet count < 100 × 10^9^/L, INR > 1.5, or an aPTT > 60 s; (f) liver dysfunction – total serum bilirubin > 40 μmol/L, and (g) serum lactate > 3,5 mmol/L. Septic shock was defined as sepsis in combination with hypotension (systolic blood pressure < 90 mmHg, or mean arterial pressure < 70 mmHg) refractory to fluid resuscitation or the requirement for vasopressors therapy to maintain adequate blood pressure.

Traditionally, septic AKI has been defined by the criteria above in accordance with the Surviving Sepsis Campaign [20]. More recent definitions of septic AKI have implemented the KDIGO or RIFLE criteria, developed for use in the nephrological setting. For compatibility with the recently published Sepsis-3 guidelines [1], the highest renal Sequential Organ Failure Assessment (rSOFA) subscore within 48 h was calculated. Renal SOFA subscore was defined as SCr (μmol/L) < 110 = rSOFA 0; 110–170 = rSOFA 1; 171–299 = rSOFA 2; 300–440 = rSOFA 3; > 440 = rSOFA 4. Patient charts were evaluated daily to screen for RRT indications during hospital-stay.

### Statistical analysis

Group comparisons in normally distributed continuous variables were tested by Student *t* test and reported as mean ± standard deviation (SD), while non-normally distributed variables were tested using the Mann-Whitney U test and reported as median and inter-quartile range. Non-normally distributed variables were log-transformed when analyzed as continuous variables. Differences in dichotomous variables were tested with the χ^2^ (chi-square) test.

Logistic regression analysis adjusted for sex and age were computed to test the relationship between penKid and selected and the following endpoints were defined: AKI development within 48 h and AKI development within 7 days, development of severe MOF and 28-day all-cause mortality. Computed odds ratios (ORs) were presented as the number of standard deviations from the mean of log-transformed penKid (z-score of ln-penKid). Odds ratios for two categorical variables were computed: as quartiles of penKid (first quartile was defined as reference) and dichotomized over a cutoff value of 100 pmol/L penKid. A 95% confidence interval was chosen. A two-sided value of *p* < 0.05 was considered statistically significant. Kaplan-Meier plot showing one-minus-survival for quartiles of penKid during 28-day follow-up was drawn for illustrative purposes. SPSS statistical software (version 25.0, SPSS Inc., Chicago, Ill) was used for all analyses.

## Results

### Characteristics of study subjects

A total of 647 patients with sepsis, i.e. fulfilment of two SIRS criteria and clinical suspicion of infection, were prospectively enrolled after presentation to the ED, of whom 588 had complete covariate data used in this study. An infectious diagnosis was made in 523 patients (88.9%). The most prevalent infections were lower respiratory tract infections (33.5%) and urinary tract infections (21.9%), followed by skin and soft-tissue infections (9.9%) and upper respiratory tract infections (9.6%). Blood cultures were drawn during the first 48 h in 536 patients (91.2%). *Escherichia coli* (*n* = 41, 7.0%), *Staphylococcus aureus* (*n* = 12, 2.0%) and *Klebsiella pneumoniae* (*n* = 8, 1.4%), were the most prevalent pathogens. A limitation of care order was issued for 90 patients (15.3%) at admission. Twenty-seven patients (4.6%) were admitted to the ICU. Two patients (0.3%) received RRT during the study period.

**Fig. 1:**
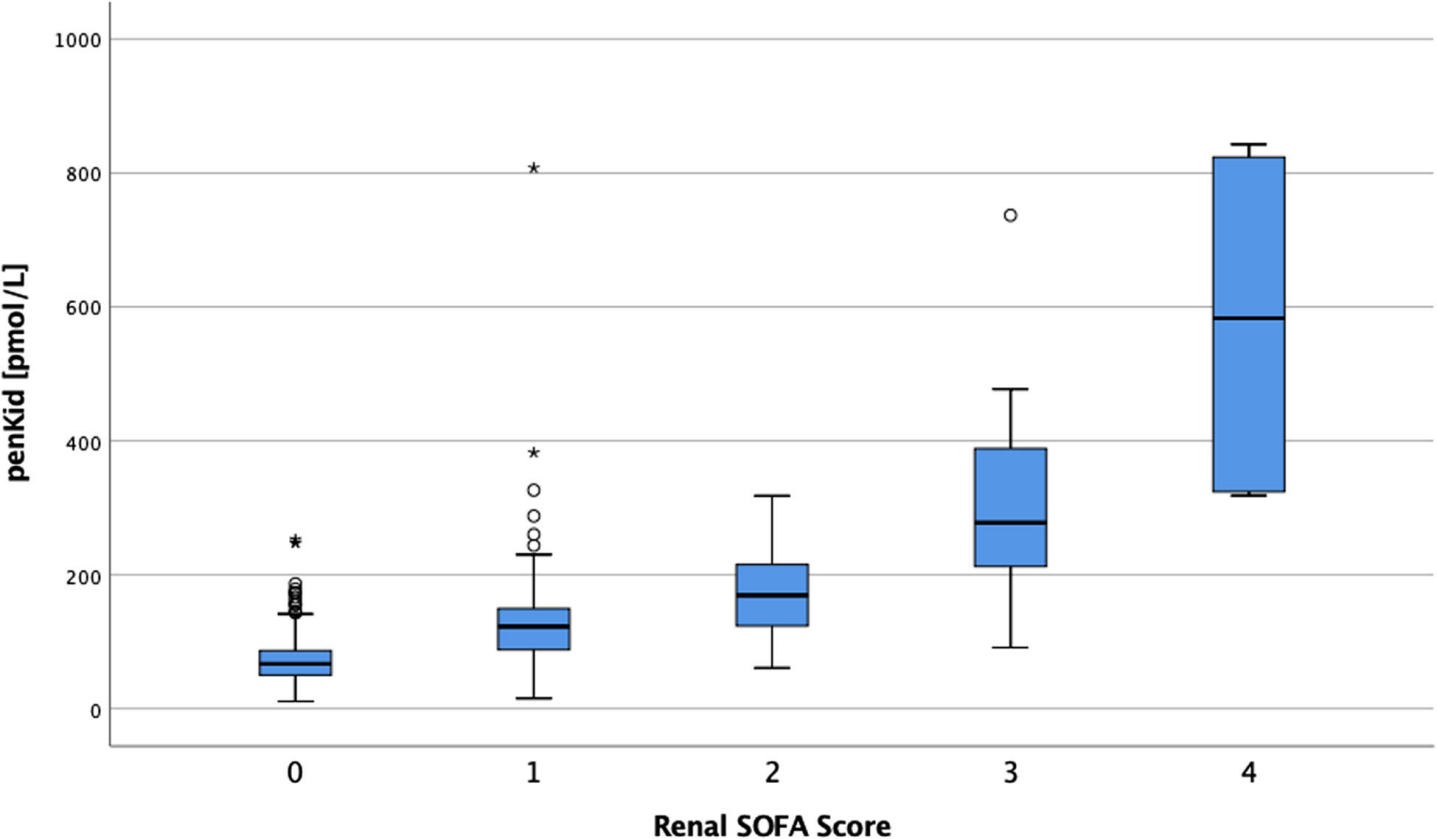
Boxplot relating renal SOFA score and Proenkephalin A 119–159 (penKid) [pmol/L] concentration in plasma

Ninety-four patients (16.0%) developed AKI within 7 days and 50 (8.5%) died within 28 days of admission. Patients who developed AKI presented with a higher burden of comorbidities compared to patients who did not develop AKI (see Table 1). Further, among those who developed AKI within 7 days, 91.4% developed severe sepsis while 17.7% suffered from septic shock versus 47.1% and 1.2% respectively in patients whose kidney function remained intact. The focus of infection was distributed similarly among patients with and without AKI (see Table 1). In patients with intact kidney function median eGFR was 73 mL/min/kg/1.73m^2^ versus 34 mL/min/kg/1.73m^2^ among those with AKI (*p* < 0.001), while SCr concentrations were 88 umol/L (no AKI) versus 155 umol/L (AKI) (p < 0.001). Median penKid plasma concentrations were 73.9 pmol/L (no AKI) versus 129.3 pmol/L (AKI) (*p* < 0.001). Distribution of penKid values across rSOFA categories is illustrated in Fig. 1. Details on patient characteristics by AKI diagnosis are presented in Table 1.

**Table 1:** Study population characteristics. Presented in amounts (percentages of total) or median value (interquartile range)

Population Characteristics	All patients (*n* = 588)	No ^a^AKI at 7 days (*n* = 494)	AKI at 7 days (*n* = 94)	*p*-value
Female sex, n (%)	288 (49.0%)	256 (43.5%)	32 (34.0%)	0.002
Age, years (IQR)	73 (61–82)	73 (59–82)	75 (67–84)	0.007
^*^CHF, n (%)	111 (18.9%)	86 (17.5%)	25 (26.6%)	0.039
^*^COPD, n (%)	110 (18.7%)	92 (18.6%)	18 (19.4%)	0.840
^*^Cancer n (%)	164 (27.9%)	133 (26.9%)	31 (33.3%)	0.198
Diabetes Mellitus, n (%)	114 (19.4%)	88 (17.8%)	26 (27.7%)	0.027
Renal Disease, n (%)	45 (7.7%)	33 (6.7%)	12 (12.8%)	0.292
^*^Immunodeficiency, n (%)	31 (5.3%)	26 (5.3%)	5 (5.4%)	0.770
^**^Limitation of care, n (%)	149 (25.3%)	110 (22.4%)	39 (41.5%)	< 0.001
Severe Sepsis, n (%)	316 (54.1%)	231 (47.1%)	85 (91.4%)	< 0.001
Septic Shock, n (%)	21 (3.7%)	6 (1.2%)	15 (17.7%)	< 0.001
Diagnosis				0.141
Pneumonia, n (%)	197 (33.5%)	169 (34.2%)	28 (29.8%)	N/A
Urinary Tract Infection, n (%)	129 (21.9%)	104 (21.1%)	25 (26.6%)	N/A
Soft-tissue Infection, n (%)	58 (9.9%)	45 (9.1%)	13 (13.8%)	N/A
^b^Other, n (%)	155 (26.4%)	134 (27.1%)	21 (22.3%)	N/A
No confirmed infection, n (%)	49 (8.3%)	42 (8.5%)	7 (7.5%)	N/A
^c^eGFR, mL/min/kg/1.73m^2^ (IQR)	66 (46–88)	73 (53–91)	34 (25–48)	< 0.001
SCr, umol/L (IQR)	88 (68–120)	80 (65–103)	155 (119–212)	< 0.001
penKid, pmol/L (IQR)	77.9 (56.9–119.7)	73.9 (53.4–101.2)	129.3 (92.2–177.5)	< 0.001

^a^Acute Kidney Injury. ^*^incomplete data, percentages do not apply to full study population. CHF, congestive heart failure. COPD, chronic obstructive pulmonary disease, diagnoses. ^**^Limitation of care order issued at presentation or during hospital stay, regarding cardiopulmonary resuscitation, intensive care, respiratory support or any combination of these. ^b^ Contains the following categories: ‘upper respiratory tract’, ‘bone’, ‘central nervous system’, ‘gastrointestinal’, ‘endocarditis’, ‘foreign body’, ‘blood port’, ‘unknown’. ^c^eGFR; estimated Glomerular Filtration Rate, mL/min/kg/1.73m^2^ calculated with the Modification of Diet in Renal Disease (MDRD) Study [18] formula

### Main results

Seventy-nine (13.4%) patients developed AKI within 48 h while an additional 15 patients (2.6%) did so > 48 h after admission but within seven days, thus totaling 94 (15.9%) AKI events at seven days after presentation. Logistic regression adjusted for sex and age yielded an OR for AKI development per z-score of log-transformed penKid of 2.5 (1.9–3.3, *p* < 0.001) and 2.5 (1.9–3.2, *p* < 0.001) for 48 h and seven days, respectively. Logistic regression of categorical quartiles of penKid concentration for AKI incidence yielded an OR of 8.5 (3.7–19.2, p for trend < 0.001) and 9.5 (4.3–20.7, p for trend < 0.001) for the highest compared to the lowest quartile (reference) at 48 h and seven days, respectively (Table 2). PenKid-based prediction of renal outcomes also remained significant across a specified cut-off of 100 pmol/L, which has previously been used to identify individuals at high-risk for poor outcome [17] [penKid > 100 pmol/L for AKI at 48 h: OR 7.3, 95% CI 4.6–13.3, *p* < 0.0001; for AKI at 7 days: OR 7.8, 95% CI 4.6–13.3, p < 0.0001].

**Table 2:** Acute Kidney Injury (AKI) at two and seven days related to Proenkephalin A 119–159 (penKid)

	All patients (*n* = 588)	*P*-value	Quartile 1 (*n* = 147)	Quartile 2 (*n* = 147)	Quartile 3 (*n* = 147)	Quartile 4 (*n* = 147)	P for trend
penKid (pmol/L)^a^	77.9 (10.9–843.0)		10.9–56.9	57.0–77.9	78.1–119.4	120.0–843.0	
AKI within 2 days							
N events (% of total) ^b^	79 (13.4%)		9 (6.1%)	6 (4.1%)	14 (9.5%)	50 (34.0%)	
OR (95% CI)^c^	2.5 (1.9–3.3)	< 0.001	Reference	0.7 (0.2–1.9)	1.7 (0.7–4.2)	8.5 (3.7–19.2)	< 0.001
AKI within 7 days							
N events (% of total)^b^	94 (15.9%)		10 (1.7%)	8 (1.4%)	17 (2.9%)	59 (10.0%)	
OR (95% CI)^c^	2.5 (1.9–3.2)	< 0.001	Reference	0.8 (0.3–2.0)	1.9 (0.8–4.3)	9.5 (4.3–20.7)	< 0.001

^a^PenKid presented as median (range), plasma concentration of proenkephalin A 119–159; OR, odds ratio; 95% CI, 95% confidence interval. ^b^N (% of total) refers to the number of participants (proportion of total number participants) with Acute Kidney Injury (AKI) events. ^c^OR (95% CI) are expressed as per z-score of log-transformed penKid and in analyses of quartiles the lowest quartile (quartile 1) was defined as the reference category and the OR (95% CI) for each of quartiles 2, 3 and 4 were compared with the reference quartile 1. Analyses were adjusted for age and sex

After adjustment for eGFR at presentation penKid-based prediction for the relationship between penKid and AKI incidence was rendered statistically non-significant for both AKI at 48 h [OR per z-score of log-transformed penKid 0.8, 95% CI 0.6–1.2, *p* = 0.327] and seven days [OR per z-score of log-transformed penKid 0.9, 95% CI 0.6–1.2, *p* = 0.448]. Mirroring logistic regression analyses for SCr for adverse kidney outcomes yielded ORs of 3.7 (2.8–5.0, *p* < 0.001) for AKI at 48 h and 3.8 (2.8–5.1, p < 0.001) for AKI at seven days (Additional file 1: Table S1). Discriminatory characteristics of penKid for AKI outcomes was determined by computing the area under the ROC-curve: AUC for AKI at 48 h penKid 0.756 (0.693–0.819), SCr 0.875 (0.839–0.912), inverted eGFR 0.866 (0.827–0.904); AKI at 7 days penKid 0.758 (0.702–0.815), SCr 0.872 (0.837–0.908), inverted eGFR 0.859 (0.821–0.898) (Additional file 2: Figure S1 and Additional file 3: Figure S2). For a summary of the relationship between other collected variables and AKI, please see Additional file 1: Table S3.

A key clinical challenge is to identify patients at risk of developing renal dysfunction and where management would have to be tailored towards a more reno-protective approach. In practice, these patients present in good condition with normal or moderately elevated SCr, which signals intact or just slightly impaired renal function which the treating physician could potentially view as insignificant in the acute management. Consequently, two subgroups of patients were defined as presenting with rSOFA 0 and no limitation of care (*n* = 359), of whom 29 (8.1%) deteriorated to rSOFA > 0, and patients presenting with rSOFA ≤1 and no limitation of care (*n* = 447), of whom 17 (3.9%) deteriorated to rSOFA > 1 within 48 h. We analysed penKid prediction for deterioration from initial rSOFA score within 48 h in patients with penKid > 100 pmol/L versus ≤100 pmol/L in these two subsets (Table 3).

**Table 3:** Worsening renal function and Proenkephalin A 119–156 (penKid) among patients with rSOFA 0 and ≤ 1

	per SD from mean of log-transformed penKid		^***^penKid > 100 pmol/L	
	^*^No eGFR adjustment	^**^eGFR adjusted	No eGFR adjustment	eGFR adjusted
^a^rSOFA = 0				
OR	2.6	1.7	5.5	3.2
(95% CI)	(1.4–4.9)	(0.9–3.2)	(2.21–13.92)	(1.1–9.1)
p-value	=0.002	=0.094	< 0.0001	=0.033
^b^rSOFA ≤ 1				
OR	3.6	2.1	10.1	3.7
(95% CI)	(1.9–6.8)	(1.0–4.4)	(3.2–31.7)	(1.0–13.1)
p-value	< 0.0001	=0.042	< 0.0001	=0.045

^*^Obtained from logistic regression model adjusted for sex, age. ^**^Logistic regression model adjusted for sex, age and eGFR, by Modification of Diet in Renal Disease (MDRD) Study [18] formula. ^***^Cutoff of 100 pmol/L has been suggested previously as significantly increased risk for renal deterioration. ^a^Presenting with an rSOFA score = 0 (intact renal function) and being up-classified to a higher rSOFA category within 48 h. Observed 29 up-classifications among 359 patients. ^b^Presenting with an rSOFA score ≤ 1 (intact and moderately impaired) renal function and being up-classified to an rSOFA category of 2 or higher within 48 h. Observed 17 up-classification among 447 patients

First, in age and sex adjusted analyses of patients with rSOFA = 0 and no limitation of care at admission, having penKid > 100 pmol/L versus ≤100 was associated with an OR of 5.5 [95% CI: 2.2–13.9, *p* < 0.0001] for deterioration from rSOFA 0 to ≥1 within 48 h of admission. After additional adjustment for baseline eGFR, prediction for rSOFA deterioration remained statistically significant for patients presenting with penKid > 100 pmol/L vs ≤ 100 pmol/L [OR 3.2, 95% CI 1.1–9.1, *p* = 0.033].

Second, among patients presenting with rSOFA ≤1 and no limitation of care at admission (*n* = 477), having penKid > 100 pmol/L vs ≤100 pmol/L conferred an OR of 10.1 [95% CI: 3.2–31.7, *p* < 0.0001] for deterioration to rSOFA ≥2 within 48 h. After additional adjustment for eGFR the relationship between penKid and up-classification to rSOFA > 1 remained statistically significant for penKid > 100 pmol/L vs ≤ 100 pmol/L [OR 3.7, 95% CI 1.0–13.1, *p* = 0.045].

We also tested if penKid > 100 pmol/L vs ≤ 100 pmol/L predicted rSOFA deterioration in all patients with rSOFA = 0 (*n* = 413) or rSOFA≤1 (*n* = 526) (i.e. irrespective of limitation of care) using a logistic regression model adjusted for sex, age and eGFR. Having penKid > 100 pmol/L vs ≤ 100 pmol/L yielded an OR of 3.5 (95% CI; 1.1–10.8, *p* = 0.027) for deterioration from rSOFA = 0 and OR = 2.1 (95% CI; 1.0–4.4, *p* = 0.044) for deterioration from rSOFA ≤1.

### Multi-organ failure and mortality

Thirty-three patients (5.6%) suffered from severe MOF. The highest penKid quartile yielded an OR of close to 30 for MOF relative to the lowest quartile (reference) and an OR of ~ 3.5 for MOF per z-score of log-transformed penKid (Table 4), an association which remained statistically significant after adjustment for eGFR [OR per z-score of log-transformed penKid 1.95, 95% CI 1.16–3.28, *p* = 0.012]. Likewise, eGFR remained predictive of severe MOF incidence in a model adjusted for sex, age and penKid [OR per mL/min/1.73 m^2^ of eGFR 0.96, 95% CI 0.94–0.99, *p* = 0.005].

**Table 4:** Proenkephalin A 119–159 (penKid) for prediction of multi-organ failure and 28-day all-cause mortality

	All patients (*n* = 588)	*P*-value	Quartile 1 (*n* = 147)	Quartile 2 (*n* = 147)	Quartile 3 (*n* = 147)	Quartile 4 (*n* = 147)	P for trend
^a^Severe Multi-Organ Failure							
^b^N events (% of total)	33 (5.6%)		1 (0.7%)	2 (1.4%)	6 (4.1%)	24 (16.3%)	
^c^OR (95% CI)	3.6 (2.5–5.3)	< 0.001	Reference	2.1 (0.2–23.0)	6.5 (0.8–55.2)	29.9 (3.8–235.3)	< 0.001
28-Day All-Cause Mortality							
N events (% of total)	50 (8.5%)		5 (3.4%)	10 (6.8%)	13 (8.8%)	22 (15.0%)	
OR (95% CI)	1.5 (1.1–2.0)	=0.009	Reference	1.3 (0.4–4.0)	1.5 (0.5–4.6)	2.2 (0.8–6.5)	=0.079

^a^Severe multi-organ failure defined as > 4 organ systems failing. Organ failure constitutes seven categories: [1] central nervous system, [2] circulatory failure, [3] respiratory failure, [4] kidney failure, [5] liver failure, [6] coagulopathy, [7] metabolic dysfunction. ^b^N events (% of total) refers to the number of participants (proportion of total number participants) for each respective endpoint. ^c^OR (95% CI) are expressed per one standard deviation (SD) increment of log-transformed penKid and in analyses of quartiles the lowest quartile (quartile 1) was defined as the reference category and the OR (95% CI) for each of quartiles 2, 3 and 4 were compared with the reference quartile. Analyses were adjusted for age, sex and eGFR calculated through the Modification of Diet in Renal Disease (MDRD) Study [18] formula

**Fig. 2:**
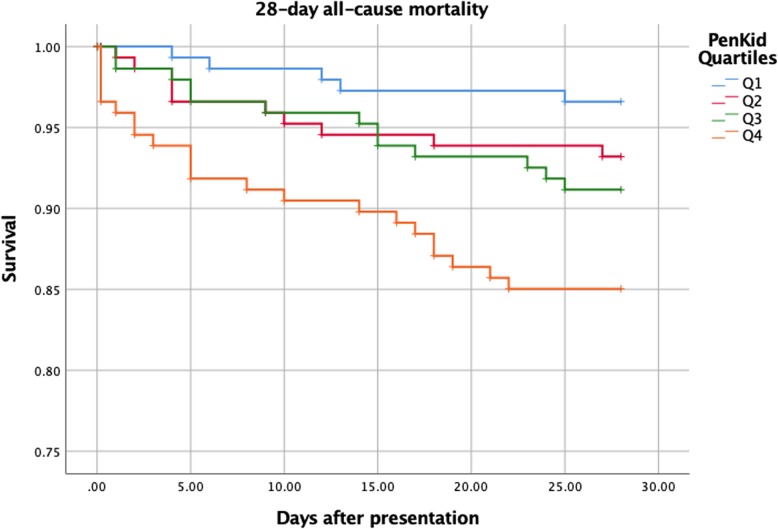
Unadjusted Kaplan-Meier plot showing 28-day all-cause mortality for quartiles of plasma Proenkephalin A 119–159 (penKid)

Fifty patients (8.5%) died within 28 days of admission with events distributed in ascending fashion across quartiles of penKid concentration illustrated in Fig. 2: quartile 1 (reference) *n* = 5, quartile 2 *n* = 10, quartile 3 *n* = 13 and quartile 4 *n* = 22. Logistic regression adjusted for age and sex yielded an OR of 1.5 (1.1–2.0, *p* = 0.009) per z-score of log-transformed penKid concentration in relation to 28-day all-cause mortality. Comparison of penKid quartiles for the same endpoint revealed a borderline statistically significant trend over quartiles (Table 4). After adjustment for eGFR, penKid remained statistically significant [OR per 1-SD increment of log-transformed penKid 1.6, 95% CI 1.1–2.3, *p* = 0.02]. In contrast, eGFR was not predictive of mortality within 28 days, neither when adjusted for age and sex [OR per mL/min/1.73 m^2^ of eGFR 1.0, 95% CI 0.98–1.00, *p* = 0.198], nor in a model adjusted for age, sex and penKid [OR per mL/min/1.73 m^2^ of eGFR 1.00, 95% CI 0.99–1.02, *p* = 0.670]. Discriminatory characteristics of penKid for organ failure and mortality outcomes was determined by computing the area under the ROC-curve (95% confidence interval): AUC for MOF penKid 0.838 (0.764–0.913), SCr 0.851 (0.782–0.921), inverted eGFR 0.857 (0.787–0.927); 28-day all-cause mortality penKid 0.676 (0.603–0.749), SCr 0.617 (0.531–0.702), inverted eGFR 0.633 (0.552–0.713) (Additional file 1: Table S3, Additional file 4: Figure S3 and Additional file 5: Figure S4). For a summary of the relationship between other collected variables and MOF and 28-day all-cause mortality, please see Additional file 1: Table S3.

## Discussion

In this prospective observational single-center study we assessed the predictive value of a single measurement of the novel biomarker penKid for AKI incidence in unselected septic patients presenting to the ED. PenKid was strongly predictive of AKI, however, not surprisingly as SCr by definition contributes to the AKI endpoint, the association between penKid and AKI was not independent of eGFR nor SCr at admission. On the other hand, penKid was independently associated with severity of MOF and, in contrast to eGFR, penKid predicted 28-day mortality. Also, among patients with normal or only slightly elevated SCr at ED admission, penKid predicted imminent renal deterioration independently of eGFR.

Patients with sepsis frequently suffer from concomitant AKI, with incidence reported to be between 5 and 20% among hospital-admissions and rising to 35–50% among the critically ill [4]. These patients suffer worse outcomes, have increased risk of in-hospital mortality and higher degree of long-term morbidity compared to patients without septic AKI [4]. Therefore, these patients require careful management and increased vigilance. However, the tools currently available to clinicians seem to be suboptimal.

In the studied cohort we observed mortality corresponding to ~ 8% at 28 days after admission and AKI incidence of ~ 16% at seven days. Similar rates of AKI incidence and mortality are presented in a recent study on septic AKI in the unselected ED setting [11], while others have shown slightly higher rates of mortality and AKI in recent publications, but these studies were carried out in the critical care setting, studying patients after ICU-admission [15, 16].

The current definition of AKI, both with and without presence of sepsis, is based on the KDIGO- or RIFLE-criteria, which include SCr and urine output. These markers have been mainstays in previous iterations of septic AKI definitions. At the time of initiation of this study, septic AKI was defined by the criteria adopted by the Sepsis-2 guidelines [20]. In practice the ED setting today, physicians almost exclusively rely on SCr to assess renal function, due to the impracticality of urine output in the acute setting. The impracticality associated with monitoring of urine output makes it difficult to rely on, especially during the primary survey in the ED. Unfortunately, both SCr and urine output are known to have limited sensitivity and specificity for AKI and oblige the clinician to follow-up with serial measurements [7]. Consequently, SCr could be deemed especially inadequate in guiding the management of septic patients with suspected AKI at the ED. [4]

The studied cohort consists of unselected septic patients in the ED. The cohort characteristics, especially regarding mortality and AKI incidence, are comparable to that of a pilot study [11] in the field of penKid prediction of AKI in sepsis. Relative to other papers studying penKid in septic AKI [15, 16], the cohort in this study could be deemed rather healthy given the fewer number of ICU-admissions and instances of RRT. It needs to be stressed, however, that these other studies were conducted in the critical care setting, following patients after ICU-admission, whereas the current study population is representative of the patients meeting the emergency physician upon immediate admission to the ED.

We found that penKid effectively predicts incidence of AKI within both 48 h of ED presentation and after seven days of admission, even if this association was not independent of eGFR at presentation. This is in fact not surprising as SCr is part of the definition of the outcome of AKI. On the other hand, a high value of penKid seems to represent a more important state of risk than a low value of eGFR, as penKid predicted 28-day mortality, whereas eGFR did not. This is in line with several previous studies [11, 15–17].

Moreover, the dependency between baseline eGFR and the AKI outcome is likely to be greatest in patients who present with high SCr. In addition, from a clinical point of view, it has been argued that AKI prediction in patients presenting with a high SCr makes little sense [16]. Thus, it can be argued that additional biomarkers are redundant in patients presenting with high SCr as the treating physician is made aware of renal dysfunction and can apply nephro-protective measures. Rather, it is among patients who present with low SCr, and impending yet clinically undetectable AKI, where the greatest benefit can be derived. Rightly so, the value of a biomarker which could effectively identify patients with a phenotype of renal impairment without clinical manifestation, so called subclinical AKI, cannot be stressed enough. Such insight could prompt the treating physician to exert higher vigilance and resort to a more nephroprotective strategy immediately upon presentation, thus optimizing prevention of renal dysfunction. As such, we believe that penKid can provide valuable insight in the monitoring of sepsis patients, especially in patients with normal SCr whose kidneys are at hidden risk. Such biomarker-guided insight can enable potentially harmful drugs, e.g. renin-angiotensin blockers and aminoglycosides, to be discontinued or dose-adjusted and thereby allow the physician to resort to a nephroprotective strategy as early as during the primary survey at the ED. The fact that penKid remained an independent predictor of renal deterioration within 48 h in patients presenting with normal (rSOFA = 0) or slightly impaired renal function (rSOFA ≤1) even after adjustment for estimated GFR indicates that penKid may improve clinical decision-making and help identify patients with subclinical AKI and also, highlights the bluntness of SCr.

Recent publications from the ICU-setting [15, 16] are suggestive of penKid being an independent predictor of poor renal outcome which adds value on top of eGFR and SCr. Clearly, these studies are conducted in the critically care setting, where patients are subject to higher-risk for poor outcome compared to that of an unselected ED population. In addition, these studies seem to suggest the added value of penKid becomes apparent when predicting for a cluster of unwanted renal outcomes, such as all-cause mortality, need for renal replacement therapy or persistent AKI, where penKid proficiently predicts the former two. Could it be that penKid prediction for AKI incidence is mainly supported by its association with mortality and need for renal replacement therapy? Surely, the most important aspect of prediction is clinical relevance, of which there is plenty of suggestive evidence for penKid.

### Strengths and limitations

The present study has a variety of strengths worth mentioning. Firstly, the studied is a genuine reflection of the real-life ED setting, including patients from all socioeconomic strata. Also, all patients were followed for a minimum of 28 days and patient records were thoroughly reviewed by infectious disease specialists to assure the correct diagnosis was made.

In summary, several patient groups at ED, as those with acute coronary heart disease or deep vein thrombosis, receive rapid and structured care, guided by the evaluation of biomarkers. Hopefully, getting the support of a biomarker that inherently raises the awareness of septic patients with risk of developing AKI, will prevent scenarios of further renal deterioration, RRT instances and will decrease serious sequelae such as chronic kidney disease and potential development of end-stage renal disease.

Nevertheless, there are also certain limitations that ought to be addressed. This study was limited to a single center, limiting applicability of the results to other EDs. In addition, the non-consecutive setting may have contributed to selection bias, as patient registration was done during office hours (6 am to 6 pm). The enrolment of patients also predates the announcement of the Sepsis-3 guidelines, even if these have been a matter of discussion within the professional community [22, 23]. An effort to maintain relevance for this updated definition of sepsis, ad-hoc analyses were performed to allow for compatibility, specifically calculation of rSOFA subscores. Our specified definition of AKI which is completely reliant on SCr in plasma and demands a degree of fluctuation, while neglecting urine output, before AKI can be diagnosed makes it difficult to evaluate the proportion of patients presenting with AKI at baseline.

Also, it is important to realise that using a SCr-based definition of AKI may not be free from significant confounders, such as diet, rhabdomyolysis, and use of nephrotoxic agents or fluid overload as a result of aggressive resuscitation.

Lastly, inulin clearance remains the golden standard method of measuring true GFR. This was neither performed nor available to our cohort of patients who were studied in the ED setting, where such a protocol would not align with the goal of providing adequate care. Further, we did not compare penKid to any other novel biomarker associated with renal dysfunction or damage, such as cystatin C or neutrophil gelatinase-associated lipocalin. One could also argue that a single measurement has limited value, where serial measurements of a predictor biomarker could better illustrate the course of a condition and prove reliability by following the clinical course. However, we argue that in the ED setting, the physician will only be helped by the initial biomarker measurement at the time of patient presentation before any therapy has been initiated.

## Conclusion

In conclusion, penKid is an effective predictor of AKI development particularly in septic patients presenting to the ED with normal serum Creatinine.

## Supplementary information

**Additional file 1: Table S1.** Acute Kidney Injury (AKI) at two and seven days related to Serum Creatinine. **Table S2.** Serum Creatinine for prediction of multi-organ failure and 28-day all-cause mortality. **Table S3.** Logistic Regression computed Odds Ratios for specified adverse outcomes for other collected variables.

**Additional file 2: Figure S1.** ROC Curve showing discriminatory characteristics of serum creatinine, penKid, eGFR for AKI within 48 h.

**Additional file 3: Figure S2.** ROC Curve showing discriminatory characteristics of serum creatinine, penKid, eGFR for AKI within 7 days.

**Additional file 4: Figure S3.** ROC Curve showing discriminatory characteristics of serum creatinine, penKid, eGFR for multiple organ failure.

**Additional file 5: Figure S4.** ROC Curve showing discriminatory characteristics of serum creatinine, penKid, eGFR for AKI within 28-day all-cause mortality.

## Data Availability

The datasets used and/or analysed during the current study are available from the corresponding author on reasonable request.

## Abbreviations

AKI: Acute kidney injury
aPTT: activated partial thromboplastin time
CI: Confidence Interval
ED: Emergency Department
eGFR: Estimated glomerular filtration rate
ICU: Intensive Care Unit
INR: International Normalized Ratio
MOF: Multi-organ Failure
OR: Odds Ratio
penKid: Proenkephalin A 119–159
RRT: Renal replacement therapy
SCr: Serum creatinine
SD: Standard Deviation
SIRS: Systemic Inflammatory Response Syndrome
SOFA: Sequential Organ Failure Assessment
