# Supplementary material for: Proenkephalin a 119–159 (penKid) – a novel biomarker for acute kidney injury in sepsis: an observational study
Source: BMC Emerg Med. 2019 Nov 28;19:75. doi: 10.1186/s12873-019-0283-9 (PMC6883703; doi:10.1186/s12873-019-0283-9)
Supplement: Supplementary file 1 — Additional file 1: Table S1. Acute Kidney Injury (AKI) at two and seven days related to Serum Creatinine. Table S2. Serum Creatinine for prediction of multi-organ failure and 28-day all-cause mortality. Table S3. Logistic Regression computed Odds Ratios for specified adverse outcomes for other collected variables. [file 12873_2019_283_MOESM1_ESM.docx]

Additional file 1

### Table S1. Acute Kidney Injury (AKI) at two and seven days related to Serum Creatinine.

|  | All patients  (n=588) | P-value | Quartile 1  (n=142) | Quartile 2  (n=152) | Quartile 3  (n=148) | Quartile 4  (n=146) | P for trend |
| --- | --- | --- | --- | --- | --- | --- | --- |
| SCr (umol/L)^a^ | 87  (68-120) |  | 57  (24-67) | 77  (68-87) | 101  (88-120) | 160  (121-981) |  |
| AKI within 2 days | | | | | | | |
| N events  (% of total) | 79  (13.4%) |  | 1  (0.7%) | 2  (1.3%) | 15  (10.1%) | 61  (41.8%) |  |
| ^*^OR  (95% CI)^c^ | 3.7  (2.8-5.0) | <0.001 | Reference | 2.1  (0.2-23.7) | 19.4  (2.5-152.9) | 131.7  (17.1-1014.9) | <0.001 |
| AKI within 7 days | | | | | | | |
| N events  (% of total)^b^ | 94  (15.9%) |  | 1  (0.7%) | 4  (2.6%) | 20  (13.5%) | 69  (47.3%) |  |
| ^*^OR  (95% CI)^c^ | 3.8  (2.8-5.1) | <0.001 | Reference | 3.9  (0.4-35.7) | 23.0  (3.0-177.6) | 133.2  (17.6-1011.0) | <0.001 |

### ^a^Serum creatinine. ^*^Odds Ratios computed through Logistic Regression adjusted for sex and age.

### Table S2. Serum Creatinine for prediction of multi-organ failure and 28-day all-cause mortality.

|  | All patients  (n=588) | P-value | Quartile 1  (n=142) | Quartile 2  (n=152) | Quartile 3  (n=148) | Quartile 4  (n=146) | P for trend |
| --- | --- | --- | --- | --- | --- | --- | --- |
| ^a^Severe Multi-Organ Failure | | | | | | | |
| N events  (% of total) | 33  (5.6%) |  | 2  (1.4%) | 0 | 5  (3.4%) | 26  (17.8%) |  |
| ^*^OR  (95% CI) | 3.4  (2.4-4-8) | <0.001 | Reference | 5.0341E^-8^  (N/A) | 3.1  (0.6-16.8) | 19.7  (4.2-92.6) | <0.001 |
| 28-Day All-Cause Mortality | | | | | | | |
| N events  (% of total) | 50  (8.5%) |  | 10  (7.0%) | 5  (3.3%) | 14  (9.5%) | 21  (14.4%) |  |
| ^*^OR  (95% CI) | 1.3  (0.9-1.7) | =0.117 | Reference | 0.4  (0.1-1.1) | 0.9  (0.4-2.3) | 1.3  (0.5-3.1) | =0.148 |

### ^*^Odds Ratios computed through Logistic Regression adjusted for sex and age.

Table S3. Supplementary Table 3. Logistic Regression computed Odds Ratios for specified adverse outcomes for other collected variables.

| Variable  ^a^Odds Ratio (95% CI)  (events/total) | Outcome | | | |
| --- | --- | --- | --- | --- |
|  | **AKI 48 H**  **(77/588)** | **AKI 7 D**  **(92/588)** | **MOF**  **(32/588)** | **28-d Mortality**  **(49/588)** |
| ^†^CHF | 1.3 (0.7-2.3) | 1.3 (0.8-2.3) | 2.2 (0.9-4.8) | 1.3 (0.7-2.6) |
| ^‡^COPD | 1.1 (0.6-2.0) | 1.0 (0.6-1.8) | 1.1 (0.5-2.6) | 1.8 (0.9-3.5) |
| Diabetes Mellitus | 1.5 (0.8-2.5) | 1.5 (0.9-2.6) | **^**^3.6 (1.7-7.4)** | 1.8 (0.9-3.4) |
| Cancer | 0.9 (0.6-1.7) | 1.1 (0.7-1.8) | 1.3 (0.6-2.9) | 1.4 (0.8-2.6) |
| Immunodeficiency | 1.0 (0.3-3.0) | 1.1 (0.4-2.9) | 2.9 (0.9-8.8) | 3.5 (1.3-9.4) |
| Renal Disease | 1.2 (0.6-2.8) | 1.7 (0.9-3.6) | 3.22 (1.3-8.0) | 1.0 (0.4-2.8) |
| Limitation of Care | **^*^2.3 (1.2-4.1)** | **^*^2.1 (1.2-3.7)** | **^***^5.9 (2.6-13.3)** | **^***^4.1 (2.1-7.9)** |

*^a^Odds Ratios adjusted for sex and age. ^†^Congestive Heart Failure. ^‡^Chronic Obstructive Pulmonary Disease. *p<0.05 **p<0.005 ***p<0.001.*
