# Supplementary figures and images for: Proenkephalin a 119–159 (penKid) – a novel biomarker for acute kidney injury in sepsis: an observational study
Source: BMC Emerg Med. 2019 Nov 28;19:75. doi: 10.1186/s12873-019-0283-9 (PMC6883703; doi:10.1186/s12873-019-0283-9)

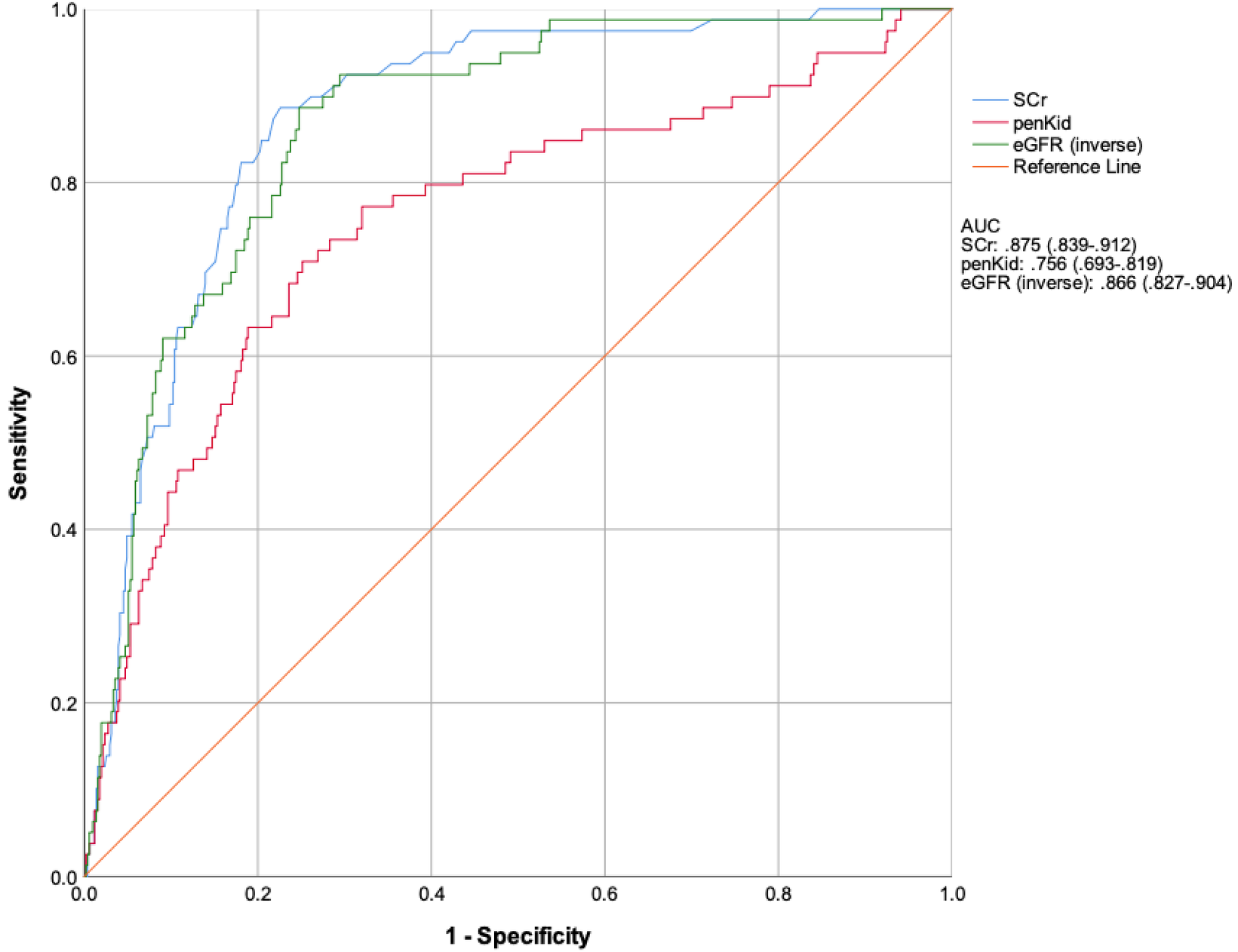

Supplement: Supplementary file 2 — Additional file 2: Figure S1. ROC Curve showing discriminatory characteristics of serum creatinine, penKid, eGFR for AKI within 48 h. [file 12873_2019_283_MOESM2_ESM.tif]

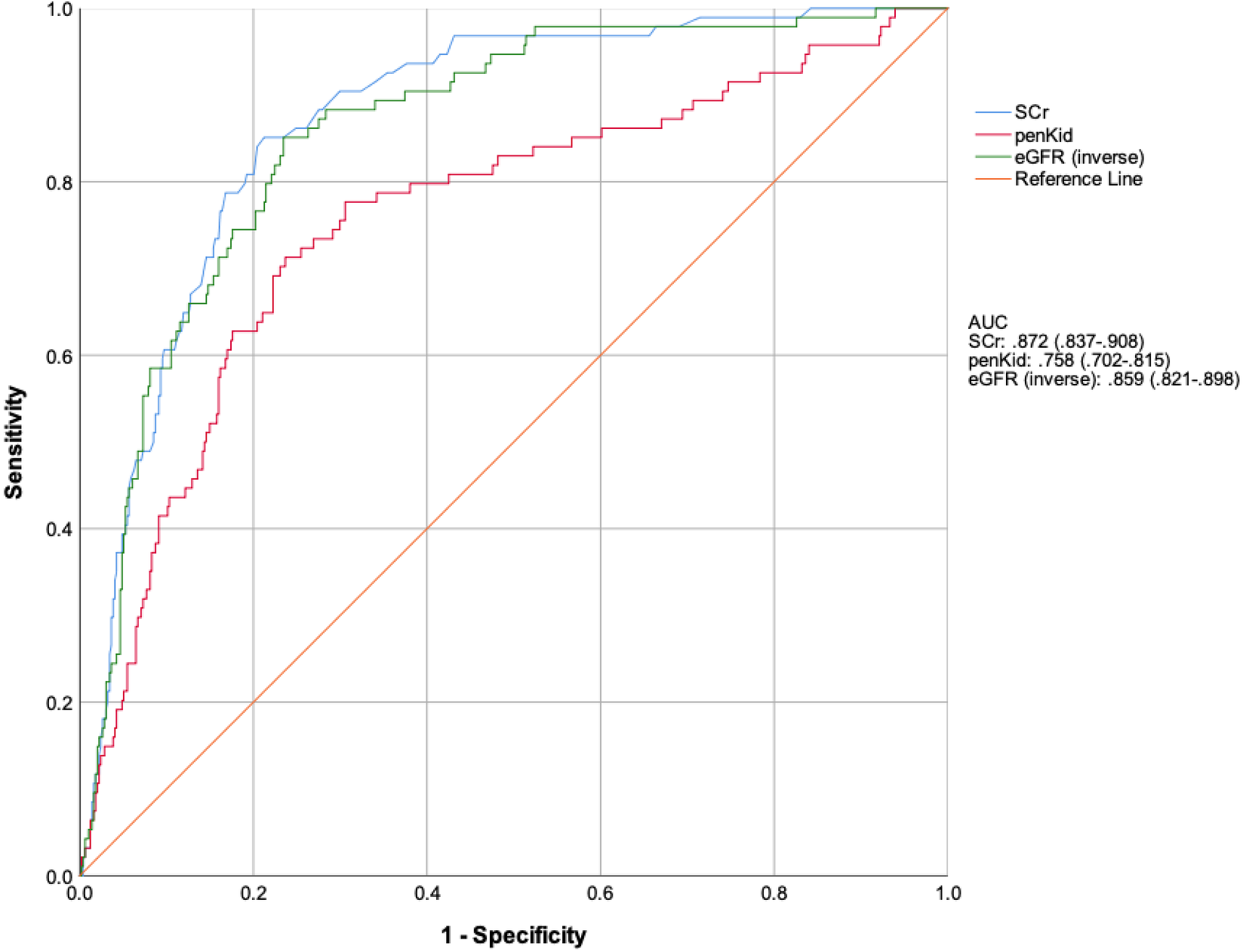

Supplement: Supplementary file 3 — Additional file 3: Figure S2. ROC Curve showing discriminatory characteristics of serum creatinine, penKid, eGFR for AKI within 7 days. [file 12873_2019_283_MOESM3_ESM.tif]

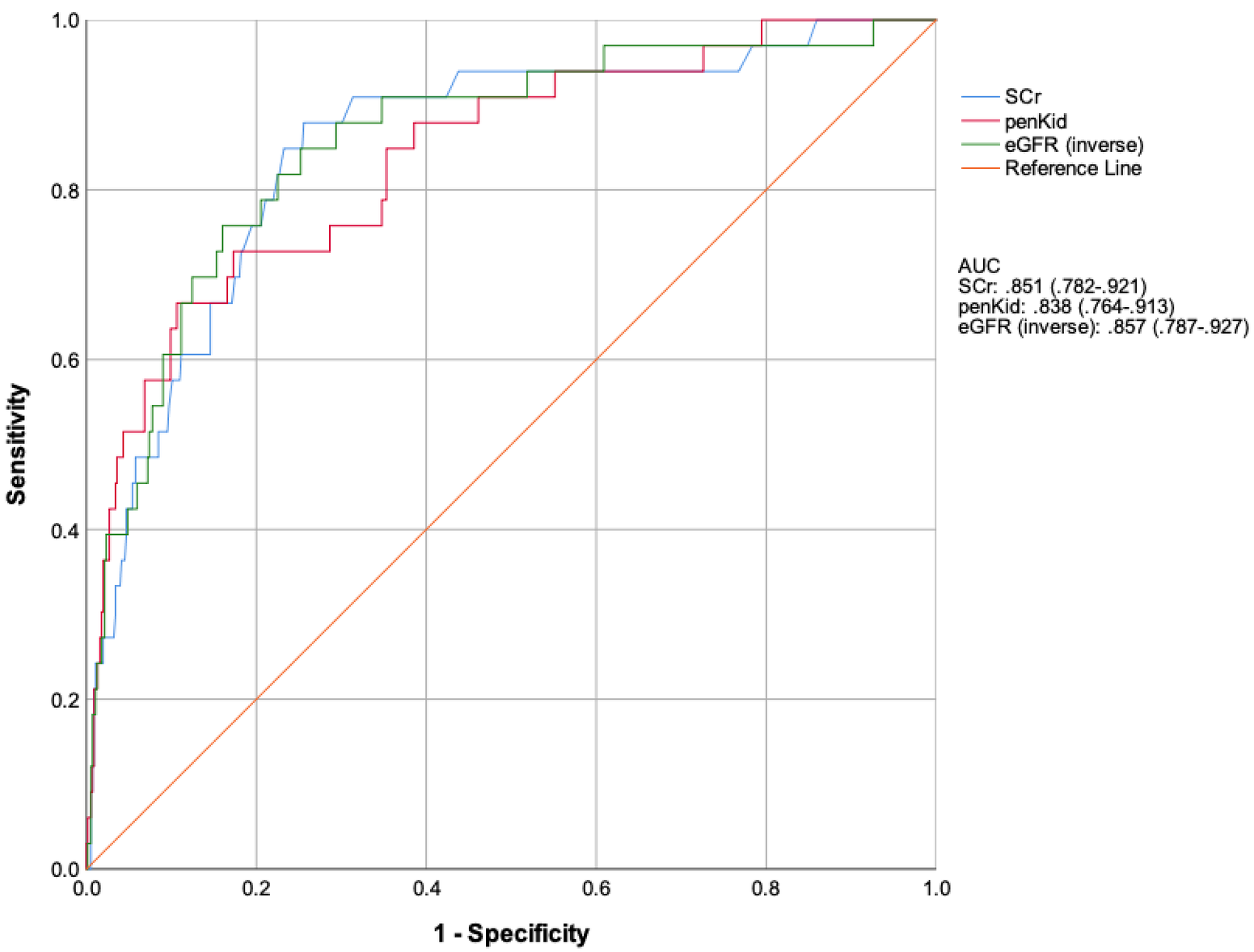

Supplement: Supplementary file 4 — Additional file 4: Figure S3. ROC Curve showing discriminatory characteristics of serum creatinine, penKid, eGFR for multiple organ failure. [file 12873_2019_283_MOESM4_ESM.tif]

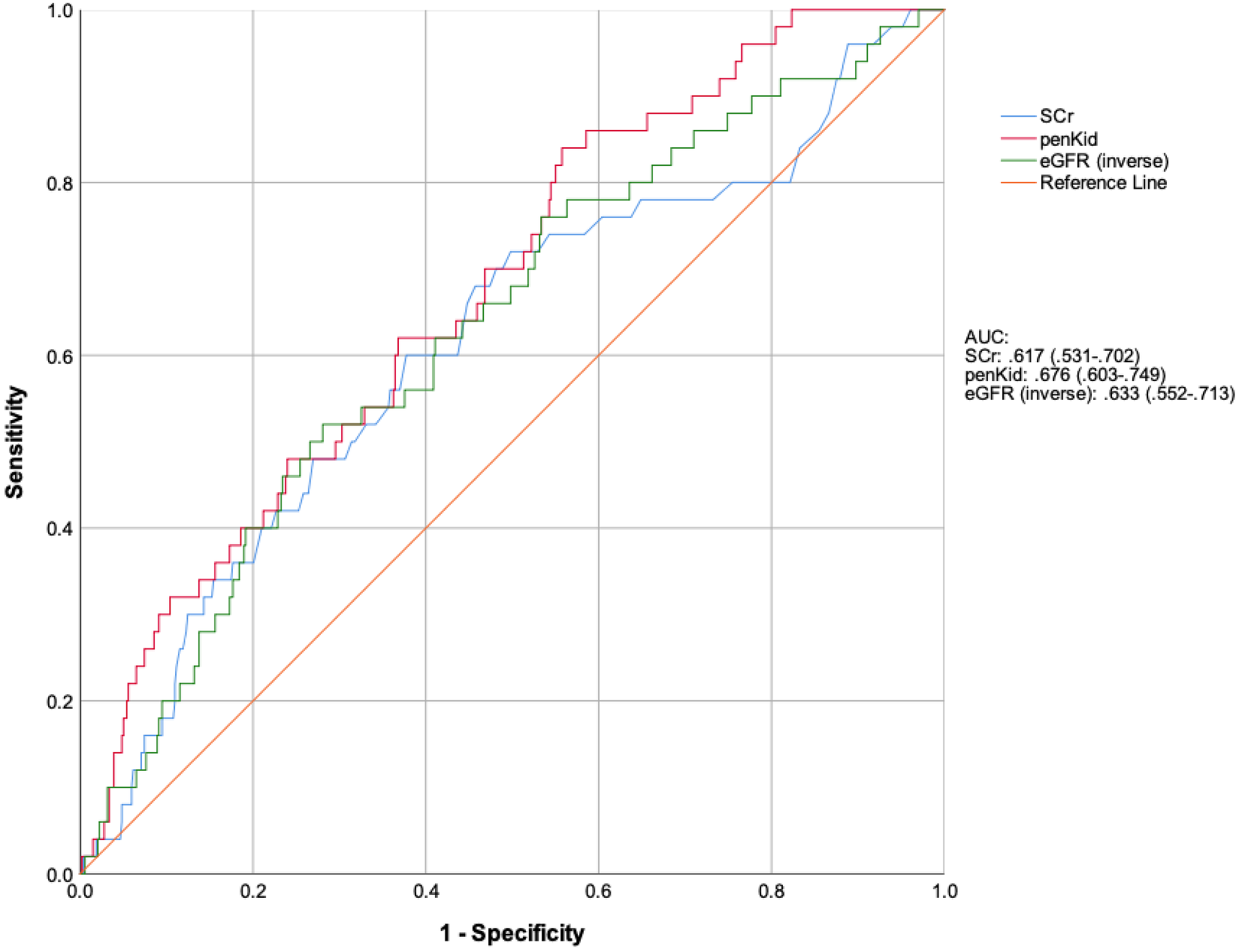

Supplement: Supplementary file 5 — Additional file 5: Figure S4. ROC Curve showing discriminatory characteristics of serum creatinine, penKid, eGFR for AKI within 28-day all-cause mortality. [file 12873_2019_283_MOESM5_ESM.tif]
